# Clinical potentials of human pluripotent stem cells in lung diseases

**DOI:** 10.1186/2001-1326-3-15

**Published:** 2014-06-17

**Authors:** Yuan Quan, Dachun Wang

**Affiliations:** 1 The Brown Foundation Institute of Molecular Medicine for the prevention of Human Diseases, University of Texas Medical School at Houston, 1825 Pressler Street/IMM 437D, Houston, TX 77030, USA

**Keywords:** Induced pluripotent stem cells, Embryonic stem cells, Differentiation and characterization, Lung stem/progenitor cells, and Lung tissue engineering

## Abstract

Lung possesses very limited regenerative capacity. Failure to maintain homeostasis of lung epithelial cell populations has been implicated in the development of many life-threatening pulmonary diseases leading to substantial morbidity and mortality worldwide, and currently there is no known cure for these end-stage pulmonary diseases. Embryonic stem cells (ESCs) and somatic cell-derived induced pluripotent stem cells (iPSCs) possess unlimited self-renewal capacity and great potential to differentiate to various cell types of three embryonic germ layers (ectodermal, mesodermal, and endodermal). Therapeutic use of human ESC/iPSC-derived lung progenitor cells for regeneration of injured or diseased lungs will have an enormous clinical impact. This article provides an overview of recent advances in research on pluripotent stem cells in lung tissue regeneration and discusses technical challenges that must be overcome for their clinical applications in the future.

## Introduction

Pulmonary diseases are one leading cause of morbidity and mortality worldwide. Currently available treatments can only alleviate symptoms or delay disease progression within a limited time range for patients with end-stage pulmonary diseases. Lung transplantation remains the only definitive treatment for many end-stage patients, yet its clinical impact is limited by shortage of suitable donor lungs and transplant rejection. More than 10% patients on waiting list die each year before transplantation [1], and the 5-year survival rate of recipients is only approximately 50% [2,3]. Recent findings in stem cell research have attracted a lot of interest to developing reliable means to regenerate injured/diseased lungs. Studies on animal lung injury models have shown that there are divergent progenitor populations responsible for maintaining homeostasis of lung epithelia in anatomically different regions. However, technical difficulty to isolate and characterize these progenitor types due to the complex lung structure continues to hinder the understanding of the role of endogenous regenerative pathways in lung tissue maintenance and repair in response to injuries. During the past several years, significant progress has been made in purification and characterization of lung progenitor cells derived from embryonic stem cells (ESCs) and induced pluripotent stem cells (iPSCs). These ESC/iPSC-derived lung progenitor cells hold great promise for understanding lung stem cell biology and disease processes, developing cell-based therapies for incurable pulmonary diseases, and generating bioengineered lungs. This review will summarize current advances in lung stem cell research and discuss technical challenges for possible therapeutic use of pluripotent stem cells in lung tissue regeneration in the future.

## Review

### Pulmonary epithelia and stem/progenitor cell types

The lung is a complex organ composed of anatomically different regions including trachea, bronchi, bronchioles and alveoli, each lined with functionally and structurally distinct epithelium. The pseudostratified epithelium covering trachea and bronchi is composed predominantly of basal, mucous secretory goblet cells and ciliated cells, along with a few discrete pulmonary neuroendocrine cells. In bronchioles, especially in the respiratory bronchioles, non-ciliated secretory Clara cells cover most of the surface area, although the less frequent ciliated cells also reside in this domain. The relative abundance of Clara cells in the transitional zone between large airways and alveoli imply that they may play an important role in coordinating the functions of the conducting airways and the gas-exchanging region. The alveolar epithelium covers more than 99% of the internal surface area of the lung and is composed of the alveolar epithelial type I cells (ATICs) and type II cells (ATIICs). ATICs are large and flat, covering about 95% of the gas-exchanging alveoli; together with endothelium of surrounding capillaries, they form a very thin blood-air interface essential for O_2_/CO_2_ exchange. In contrast, ATIICs are small, cuboidal cells, and play an important role in maintenance of alveolar homeostasis.

As pulmonary epithelia are constantly exposed to injurious stimuli from the environment, the lung needs to generate new cells to replenish injured and aged epithelial cells for maintaining normal structure and function. It is known that the endogenous repair capacity of the lung is relative low [4-7] and may fail after repeated challenges, leading to development of life-threatening pulmonary diseases such as asthma, chronic obstructive pulmonary disease, and idiopathic pulmonary fibrosis. Due to lung structural complexity with cellular diversity and slow epithelial cell turnover rates, it is still a challenge to isolate and characterize lung stem/progenitor cells for exploring the lung disease processes and endogenous repair mechanisms. To date, whether there are undifferentiated multipotent stem cells with the ability to self-renew indefinitely in lung is controversial. Injuries to mobilize stem cells for tracking DNA label-retaining cells (LRCs) are a key approach to identifying stem/progenitor cell compartment in distinct lung regions. There is mounting evidence showing that local progenitor cells function to renew injured and aged epithelial cells in anatomically different regions [8-11]. In the trachea and larger airways, basal cells have traditionally been considered as progenitor cells, with the capacity for proliferating and differentiating into basal, ciliated, and goblet cells [12,13]. In human, the large airway epithelium is more pseudostratified, and there are parabasal cells located right above the basal cells. Because parabasal cells express high level of proliferation marker MIB-1, they are thought to function as transient amplifying (TA) cells derived from basal cells in the larger airways [14,15]. In addition, subcutaneous transplantation experiments have shown that tracheal grandular cells can repopulate tracheal epithelium. In SO_2_-induced tracheal injury model of mice, LRCs accumulated in submucosal gland ducts lasted longer than those in tracheal epithelium [16,17], suggesting that submucosal gland ducts may harbor relatively primitive precursor cells for tracheal epithelium.

In distal lung, injury studies using naphthalene, a Clara cell-specific toxic agent, to deplete Clara cells have revealed that two populations of progenitor cells are responsible for the maintenance of bronchiolar epithelium: the abundant facultative TA Clara cells, and a subset of variant Clara cells that is cytochrome P450 2 F2 negative and stem cell antigen positive [18-22]. These variant Clara cells are naphthalene-resistant and express Clara cell-specific marker CCSP. They reside in discrete groups at the bronchioalveolar duct junction (BADJ) and the branch point-associated neuroepithelial bodies. It has been demonstrated that the variant Clara cells located at BADJ also express ATIIC-specific surfactant protein C (SPC) [23,24]. These cells appear to possess the ability to proliferate and differentiate into both bronchiolar and alveolar epithelial cells *in vitro*, and may be important in the maintenance of both bronchiolar and alveolar epithelial cell populations [23]. Studies using mouse injury model have shown that ATIICs possess proliferative capacity and play an important role during the re-epithelialization of alveoli after lung injury. In addition, multipotent stem cells with the potential of generating distal lung-like tissue *in vivo* have been isolated from adult lungs of sheep and rats [25]. More recently, undifferentiated human lung stem cells (hLSCs) were identified in distal lungs. These cells are antigen c-kit^+^ and have the capacity to generate human bronchioles, alveoli and pulmonary vessels after injected into injured mouse lungs [26]. These studies provide strong evidence for the existence of distal lung stem cells, yet their relationship with Clara and ATIICs is still unclear.

### Lung epithelial progenitor cells derived from ESCs

#### Strategies to derive ESCs into lung epithelial progenitor cells

The ESCs isolated from the inner cell mass of blastocyst-stage embryos are undifferentiated, pluripotent cells [27,28], and can be induced to differentiate *in vitro* into a wide range of different cell types [29-36]. ESC-derived lung stem/progenitor cells are a promising cell source for exploring therapeutic methods treating lung injuries and pulmonary genetic disorders. Due to the fact that most lung stem/progenitor cell types as well as their hierarchy have not been well characterized, progress in development of procedures to derive ESCs into lung stem/progenitor cells has been slow, and the mechanisms underlying the differentiation of ESCs into large lung airway epithelial cells remains elusive. It has been demonstrated that ESCs can be differentiated into Clara cells [37,38] and ATIICs via embryonic body (EB) formation or co-culture with pulmonary mesenchyme [39-41]. However, these spontaneous differentiation procedures are not efficient, generating only a very small percentage of ESC-derived lung cells [42]. Recently, procedures to enrich definitive endoderm for effective differentiation of ESCs into ATIICs have been developed by using a growth factor cocktail or a lung-specific cell-conditioned medium [43], but are not yet successful in generating a homogenous population of ATIICs. In these published studies, the derivation of ATIICs from ESCs was demonstrated by ATIIC-specific SPC expression and morphological appearance of lamellar bodies. It remains unclear whether these ESC-derived ATIICs possess normal biological function. In addition, these differentiated cultures generate a mixed population of cell derivatives and may contain the remaining pluripotent cells, which is not suitable for transplantation because they carry a significant risk of producing teratomas after transplantation *in vivo*.

In order to obtain transplantable ESC-derived ATIICs, our laboratory has developed a reliable procedure to generate stably transfected human ESC (hESC) lines containing a single copy of ATIIC-specific SPC promoter/neomycin^R^ (NEO^R^) transgene [44]. As SPC is specifically expressed by ATIICs, only hESC-derived ATIICs can express NEO^R^ and survive in G418 selected cultures. Without the need of EB formation, these hESC lines can be selectively differentiated into an essentially pure population of ATIICs (hES-ATIICs) when cultured on matrigel coated dishes in the presence of G418. These hES-ATIICs show the biological features and morphological characteristics of normal ATIICs, including lamellar body formation, expression of surfactant proteins, and the ability to proliferate and differentiate into ATICs *in vitro.* Hence, they can serve as a transplantable source of ATIICs for the exploration of their possible clinical application in the future [45].

#### Characterization of ESC-derived ATIICs *in vivo*

Bleomycin (BLM)-induced alveolar injury is well characterized in mice [46,47], and has been used as a model to test therapeutic potential of hES-ATIICs [45]. When transplanted into SCID mice on day 1 or 2 after exposure to BLM, a substantial number of hES-ATIICs remained in the injured lungs; approximately 34% of these expressed ATIC markers on day 10 after BLM challenge, suggesting that these transplanted hES-ATIICs had differentiated into ATICs *in vivo*[45]. Lung injury was abrogated in mice transplanted with hES-ATIICs, demonstrated by significantly decreased collagen deposition and complete recovery of body weight and arterial blood oxygen levels. It was also noted that numerous endogenous mouse ATIICs survived in the BLM-injured lungs of hES-ATIIC transplanted mice, but not in BLM-injured lungs receiving monocytes or normal saline, suggesting that structurally engrafted hES-ATIICs may provide an appropriate signal microenvironment via their paracrine effect in activating endogenous repair. Thus, structural engraftment and differentiation of hES-ATIICs coupled with activated endogenous ATIICs can lead to robust re-epithelialization of injured alveoli. Further investigations are needed to characterize the signaling communication between ATIICs for maintaining alveolar homeostasis. We also found that BLM-injured mice transplanted with hES-ATIICs survived as normal mice without experiencing tumorigenic side effects, demonstrating that genetic selection is an effective approach to generating transplantable ESC-derived lung stem/progenitor cells. However, random transgene insertion into the chromosomal DNA may cause unpredicted critical gene dysfunction. In addition, only approximately 5% hES-ATIICs remained in the BLM-injured lungs 10 months after transplantation. Poor long-term retention/survival of engrafted hES-ATIICs in injured lungs of SCID mice [45] indicates that the mouse lung may reject hES-ATIICs or fail to provide an appropriate microenvironment for hES-ATIICs to rebuild the progenitor cell pools after injury. Therefore, mouse ESCs (mESCs) should be used instead to explore the potential long-term therapeutic benefit of ESC-derived ATIICs in mice injury models. More recently, our laboratory has generated genetically modified mESC lines harboring a single copy of ATIIC-specific NEO^R^ transgene in Rosa 26 gene locus [48]. These random genetic insertion-free mESC lines can be directed to differentiate into a homogenous population of functional ATIICs, providing a reliable source of cells for further characterization of ESC-derived ATIICs in mice lung injury models.

### Lung epithelial progenitor cells derived from iPSCs

The methodology for reprogramming of somatic cells into iPSCs with defined reprogramming factors (*Oct4, Sox2*, *cMyc* and *Klf4*) was established by Yamanaka’s group in 2006 [49]. As renewable source of autologous, disease-specific cells, iPSCs hold great promise in regenerative medicine for modeling diseases, screening drugs and developing cell-based therapeutic strategies. Initially, the technique uses retrovirus or lentivirus to deliver necessary reprogramming factors for successfully reprogramming [49-51]. However, as random integration of multiple copies of each transgene to over-express reprogramming factors for reprogramming is a stochastic process, the generated iPSC lines exhibit considerable phenotypic variation and may have unpredictable critical gene dysfunction. In addition, constant expression of reprogramming transgenes may result in deleterious outcomes in which the derived iPSCs could develop into tumor or lose their pluripotency. For safety improvement, many alternative techniques have been developed by using non-integrating vectors [52-55] or PiggyBac transposon system [56] to avoid or eliminate insertional mutagenesis, yet the application of these methods is limited by the impractically low reprogramming efficiency. Recently, iPSCs have been generated by direct delivery of recombinant proteins, RNAs, or microRNAs [57-59]. These nongenetic techniques allow generating genetic integration-free iPSCs, yet they require special expertise for preparing proteins, RNA and mature microRNA and need multiple times of transduction/transfection for reprogramming, and the reprogramming process is still stochastic with low reprogramming efficiency. Even though efficient generation of genetic integration-free and exogenous reprogramming-factor-free iPSCs continues to be a challenge, a series of animal studies have demonstrated the potential clinical value of iPSC-derived progenitor cells in treatment of congenital hypomyelination, spinal cord injury, diabetes, and Parkinson’s disease [60-63]. Recently, human lung disease-specific iPSC lines associated with either cystic fibrosis or alpha-1 antitrypsin deficiency-related emphysema have been generated with a single excisable lentiviral vector [64]. Although the derived iPSC lines could be useful for lung disease modeling, the use of a viral vector for reprogramming and the part of DNA sequence left behind at unknown site(s) after removal of transgenes are potential drawbacks. In addition, difficulty to obtain iPSC derived transplantable cell types is still a major obstacle to developing clinical application of iPSCs.

**Figure 1:**
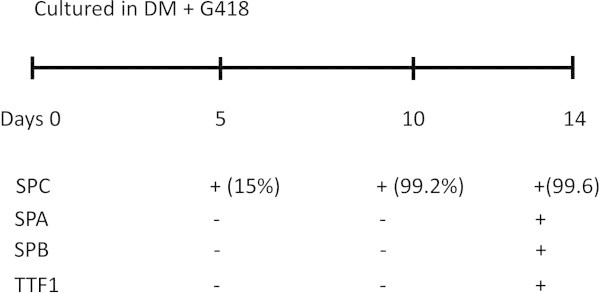
**Diagram of hiPSC differentiation.** The hiPSCs were cultured on matrigel-coated plates in DM containing 20 μg/ml of G418 for 14 days. The SPC positive cells were observed in the differentiating cultures at a very early time point (day 5), and can be enriched into a homogenous population (>99%) with robust proliferation capacity on day 10. The hiPSC-derived ATIICs were observed in the cultures on day 14, which expressed TTF1 and surfactant proteins (SPA, SPB, and SPC).

**Figure 2:**
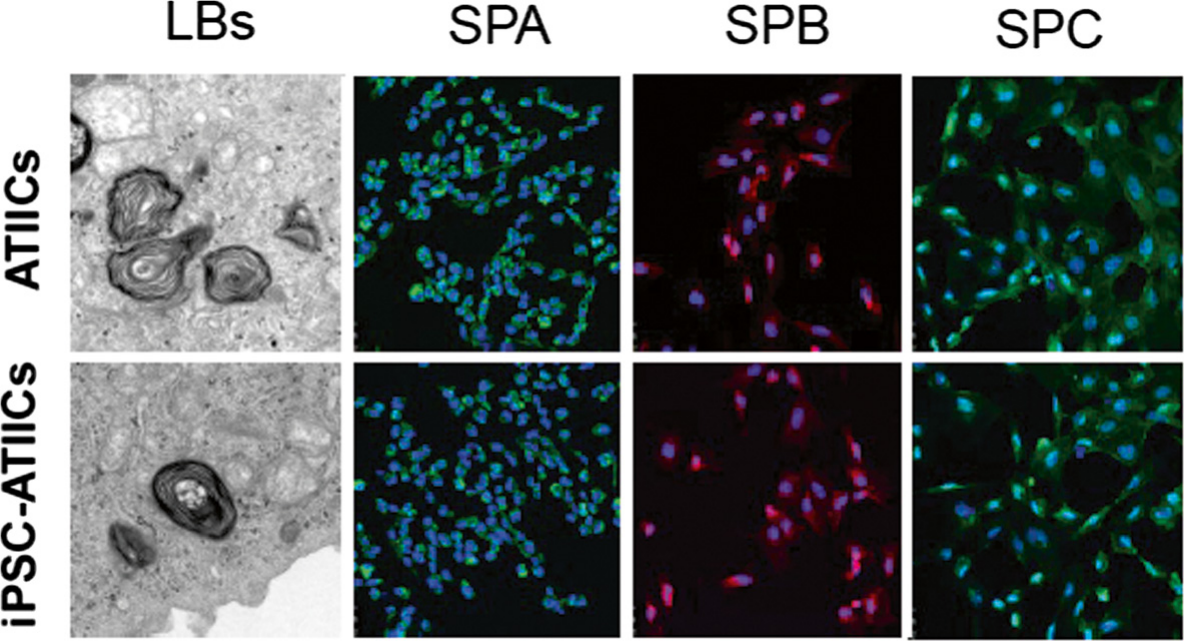
Electron micrographs showed well-developed lamellar bodies (LBs) in G418-selected hiPSC-ATIICs, as in the control human ATIICs; the immunofluorescent staining images demonstrated that G418-selected hiPSC-ATIICs expressed SPA, SPB and SPC as control human ATIICs did.

In order to generate random-insertion-free and exogenous reprogramming-factor-free hiPSCs that can be selectively differentiated into pure populations of ATIICs without the need of any additional treatment or genetic modification, our laboratory developed a single site-specific targeting vector, which harbors the reprogramming transgenes controlled by Tet-On inducible gene expression system, ATIIC-specific NEO^R^ transgene, and loxP targeting sequence [65]. With this non-viral targeting vector, all required transgenes can be inserted into a site immediately downstream of β-2-microglobulin (B2M) gene locus without causing B2M dysfunction. The reprogramming transgenes can be induced to express for efficient reprogramming of human skin fibroblast, and subsequently removed to obtain genetic integration-free and exogenous reprogramming-factor-free hiPSC lines. Because the derived hiPSCs contain the ATIIC-specific NEO^R^ transgene, they can be selectively differentiated into an essentially pure population of functional ATIICs after cultured on matrigel-coated dishes in differentiation medium (DM) with 20 μg/ml of G418 for 14 days [65]. The selective differentiation procedure for generation of hiPSC-derived ATIICs is illustrated in Figure 1. The SPC^+^ cells can be enriched as early as on day 10. It was found that these early derived SPC^+^ cells did not express surfactant protein A (SPA) and B (SPB), but possessed robustly proliferative capacity, suggesting that they may be early primitive progenitor cell type(s). On day 14, the hiPSC-derived SPC^+^ cells (hiPSC-ATIICs), which did not express CCSP, expressed thyroid transcription factor one (TTF1) and surfactant proteins with typical ultra-structure of ATIICs (Figure 2). We showed that these hiPSC-ATIICs possess regulatable capacity for synthesizing and secreting surfactant proteins and lipids as primary human ATIICs. Like hES-ATIICs discussed above, the hiPSC-ATIICs have great potentials in engrafting and differentiating into ATICs *in vivo* for re-population of BLM-injured alveoli and repair of damaged pulmonary function. This is a one-step site-specific genetic modification. This novel strategy makes it possible for the first time to generate lung disease-specific hiPSC-ATIICs for exploring their clinical application and modeling alveolar diseases.

### Lung tissue bioengineering

Proof-of-principle studies in tissue engineering have strongly demonstrated the potential of engineered functional organs such as heart, liver and kidney [66-68]. Generation of engineered lungs is an attractive prospect for the treatment of end-stage pulmonary diseases. Several groups have been able to culture fetal and adult lung cells in three-dimensional culture systems with synthetic scaffolds for creating engineered lung tissue *ex vivo*[69-73]. While the natural and synthetic scaffolds are capable of supporting differentiation of ESCs into CCSP^+^ and SPC^+^ cells, it is not clear whether these cells are functional distal airway and alveolar cell types. The published data have shown the potential of Gelfoam sponge scaffold seeded with fetal rat lung cells in forming alveolus-like structure *in vivo*[73]. However, whether the engineered tissue is capable of ventilation-perfusion for gas exchange remains to be investigated.

Recently, substantial advances have been made in engineering functional trachea using an artificial matrix and a decellularized, cadaveric scaffold [74,75]. Macchiarini et al. developed a tissue-engineering procedure to generate a trachea by seeding recipient’s airway epithelial cells and chondrocytes onto a decellularized, cadaveric trachea scaffold within a custom bioreactor. The engineered airway segment was then used to replace the left main bronchus with malacia to restore the end-staged patient’s pulmonary function without the need of immunosuppressive drugs. The 5-year follow-up results showed that the patient had a normal life, suggesting that the tissue-engineering strategy is safe and promising in generating engineered trachea for autologous transplantation [76].

It is intrinsically difficult to decellularize and recellularize whole lung tissue to recapitulate its complex structure. Despite this, significant progresses have also been made in using a whole decellularized lung as a scaffold to generate engineered lung [77,78]. The fully decellularized lung retains its native architecture with acellular vasculature, airways and alveoli, and appears superior to other simple bioartificial scaffolds to support differentiation and recellularization of pluripotent stem cells. The cultured lung epithelial and vascular endothelial cells in the decellularized lung scaffold can efficiently repopulate the epithelial and vascular structures in a custom bioreactor to generate engineered lung with the potential for ventilation and gas exchange *in vivo*. However, due to the fact that some of decellularized areas are not completely recellularized to reconstitute the alveolar-capillary barrier, there are red blood cells and thrombus formations in the airspaces of engineered lungs after transplantation. Although the engineered lungs participate gas exchange *in vivo* for only short time intervals, these significant advances demonstrate the feasibility of using whole decellularized, cadaveric lungs as scaffolds for generation of functionally engineered lungs for transplantation.

Using patient-derived iPSCs to recellularize the decellularized, cadaveric lung scaffolds that lack donor cells expressing alloantigens is expected to generate functionally engineered lungs for “autologous” transplantation. Like ESCs, iPSCs have the potential to differentiate into many different tissue cell types. Thus, preventing nonspecific differentiation and overgrowth of iPSCs will be a challenge for generating engineered lungs. Recently, the relatively primitive c-kit^+^hLSCs have been identified. The c-kit^+^hLSCs are lung-specific and have the capacity for generating distal lung structure, including bronchioles, alveoli and blood vessels *in vivo*[26]. Although the ability of c-kit^+^hLSCs to generate larger airway still remains to be investigated, hiPSC-derived c-kit^+^hLSCs is considered a promising source of cells that can be used to recellularize the decellularized lung scaffold. Developing a strategy to direct differentiation of patient-derived iPSCs into the more primitive c-kit^+^hLSCs that can be used to recellularize the decellularized lung scaffold to reconstitute its complex structure with orchestrated and coordinated epithelia and endothelium is an attractive prospect.

## Conclusion

Potential application of lung stem/progenitor cell types derived from iPSCs and ESCs holds great promise in modeling pulmonary diseases and developing cell-based therapies for treatment of severe lung diseases. Because ESCs and their derivatives express alloantigens, including MHC class I molecules that are primary targets for immune rejection by self-recognizing T cells, development of immune rejection towards grafted ESC derivatives is a major limitation to their clinical application. Patient-derived iPSCs are conceivably genetically identical to the patient. However, reprogramming may result in a number of abnormal gene expressions in iPSC-derived cells, causing immune rejection in the syngeneic recipients as reported by Zhao et al. [79]. Technologies for generating iPSCs need to be further improved in order to obtain “clinical grade” iPSCs. Whether the novel strategy that we have developed to generate the genetic integration-free and exogenous reprogramming factor-free hiPSC lines may cause abnormal gene expression or defects needs to be further evaluated. As ESCs are reliable and versatile for generating many different tissue cell types, it is an appealing option to generate a “universal” hESC line with significantly reduced expression of MHC molecules. It is hoped that lung stem/progenitor cells derived from a “universal” hESC line can be transplanted without evoking robust immune response from HLA class I mismatched recipients, and thus can be used to generate engineered lungs for clinical use in the future.

## Competing interests

The authors have no financial conflict of interests to declare.

## Authors’ contributions

YQ and DW contributed equally to the literature review and manuscript preparation. Both authors read and approved the final manuscript.

## Authors’ information

Y.Q is a research fellow, and D.W. an assistant professor in The Brown Foundation Institute of Molecular Medicine for the prevention of Human Diseases, University of Texas Medical School at Houston, Houston, TX 77030, USA. The major research goal of our project is to explore potential application of lung stem/progenitor cell types derived from iPSCs and ESCs for modeling pulmonary diseases and developing cell-based therapies for the treatment of severe lung diseases in animal models.
